# Optimal Parameter Exploration for Online Change-Point Detection in Activity Monitoring Using Genetic Algorithms

**DOI:** 10.3390/s16111784

**Published:** 2016-10-26

**Authors:** Naveed Khan, Sally McClean, Shuai Zhang, Chris Nugent

**Affiliations:** 1 School of Computing and Information Engineering, Ulster University, Coleraine, Co., Londonderry BTT52 1SA, UK; si.mcclean@ulster.ac.uk; 2 School of Computing and Mathematics, Ulster University, Jordanstown, Co., Antrim BT37 0QB, UK; s.zhang@ulster.ac.uk (S.Z.); cd.nugent@ulster.ac.uk (C.N.)

**Keywords:** multivariate change detection, activity monitoring, multivariate exponentially weighted moving average, accelerometer, genetic algorithm, change-point detection

## Abstract

In recent years, smart phones with inbuilt sensors have become popular devices to facilitate activity recognition. The sensors capture a large amount of data, containing meaningful events, in a short period of time. The change points in this data are used to specify transitions to distinct events and can be used in various scenarios such as identifying change in a patient’s vital signs in the medical domain or requesting activity labels for generating real-world labeled activity datasets. Our work focuses on change-point detection to identify a transition from one activity to another. Within this paper, we extend our previous work on multivariate exponentially weighted moving average (MEWMA) algorithm by using a genetic algorithm (GA) to identify the optimal set of parameters for online change-point detection. The proposed technique finds the maximum accuracy and *F_measure* by optimizing the different parameters of the MEWMA, which subsequently identifies the exact location of the change point from an existing activity to a new one. Optimal parameter selection facilitates an algorithm to detect accurate change points and minimize false alarms. Results have been evaluated based on two real datasets of accelerometer data collected from a set of different activities from two users, with a high degree of accuracy from 99.4% to 99.8% and *F_measure* of up to 66.7%.

## 1. Introduction

The current enhancements in wireless communication and processor technologies have empowered the deployment of low cost, power efficient, and small sensor nodes in different domains such as education, industries, and healthcare [1,2]. In these scenarios, one of the key considerations is how to highlight and monitor events of interest. Additionally, smart monitoring is an important application of sensor networks and has received increased attention during the last few decades [3]. The complex and changing nature of human activities are often vague with regard to which information is more significant to identify activities. Activity recognition has a number of important applications in ambient assisted living. The interactive hospital (iHospital) [4] has been equipped with smart devices to automatically recognize user activities and provide services to hospital staff. Contextual information is processed using a hidden Markov model to recognize user activities. Likewise, radio frequency identification (RFID) technology [5] has been used to localize elderly patients affected by dementia. RFID technology provides help to patients and medical professionals but may compromise patient privacy. Moreover, activity monitoring is a fundamental aspect of context-aware systems for identifying users to solicit activity labeling after switching to a new activity [6] or to identify and detect changes in a patient’s vital signs [7]. The main objective of such systems within healthcare is to detect activities of daily living and to monitor these over time. The activities can be periodic actions such as “walk”, “stand”, “run”, “sit”, and so forth. Recently, wearable sensors like accelerometers or gyros have become smaller in weight and size and have been embedded into many types of wearable devices, smart phones, and smart watches. Moreover, these tiny, fast-processing, large-memory-storage, and efficient (low power) communication sensors [6] can help in data collection. These wearable sensors are widely used to capture and identify different transitions of movement patterns for various periodic activities [8]. Change-point detection is used to classify the transition from one underlying time-series generation model to another. The abrupt variation in mean, variance, or both may represent change in time-series data. In time-series data, the best change-point detection methods have used probability distributions for comparison of past and current intervals. Additionally, numerous methods have used an explicit strategy to prompt an alarm for a particular change point when two distributions become significantly different [7,9]. Moreover, the timely and precise pattern extraction and prediction from observed data is essential in numerous decision-making systems. However, the varying nature of data models presents immense challenges for learning algorithms and data-mining techniques [10]. Change-point detection can be classified as online or offline. In offline detection, the data is collected first and then the change point algorithm is used to collectively process all the data at once. However, online change-point detection algorithms are used in real-time systems to observe, monitor, and evaluate data simultaneously as it becomes available. Such algorithms need to be fast, sequential, and minimize false alarms.

However, automatic change-point detection for the purpose of activity recognition is still a challenging research task. Also of importance is the choice of a lightweight algorithm to be implemented in an online detection scenario to automatically detect the change point in user activities. In various situations, the timely response must be expedient—for example monitoring a patient’s vital signs, such as observing heart rate during different activities—and also able to generate real world annotated datasets by annotating the activities [6]. A manual activity-labeling task requires significant amounts of time and labor, and it remains an obstacle to formulate activity-recognition systems with ease. 

In this paper, we extend our previous work for change point detection using multivariate exponentially weighted moving average (MEWMA) [11]. The MEWMA approach is used to measure more than one characteristic of a system and also to evaluate the relationships among these characteristics. The advantage of using MEWMA is to analyze all the covarying time-series at the same time thus taking into account interrelationship between the variables. MEWMA is used with standard and tuned parameters such as λ, which weights the current versus historical data, window size and significance values with the aim of change-point detection. Also, the MEWMA approach tunes the different parameters to achieve better performance and accurate change-point detection. The limitation of the previous approach is that each parameter set needs to be evaluated manually to find the optimal parameter set, which makes the approach computationally intense. In this paper, a genetic algorithm is proposed to automatically identify an optimal parameter set, using a fitness function for MEWMA, using parameters such as the forgetting parameter λ, the window size, and significance value for each activity so as to maximize the *F_measure*. The *F_measure* is used as a measure to find the overall effectiveness of the activity recognition by combining the precision and recall. A genetic algorithm is used to mimic the process of evolution by taking a population of strings, which encodes possible solutions, and combining them based on the fitness function to produce solutions that are high performing [12]. The remainder of this paper is structured as follows. Section 2 presents an overview of background work specific to change-point detection. In Section 3 we provide an overview of MEWMA and genetic algorithms (GA). The experimental setup with results is presented in Section 4. Finally, conclusions and future work are presented in Section 5.

## 2. Background

Online change detection can be used in real-time scenarios that can be analyzed as soon as data becomes available. The varying nature of input data creates substantial challenges for numerous learning algorithms. The timely and precise pattern extraction and prediction from observed data is essential for decision-making systems. Thus, the most important issue that still needs to be addressed is the accurate and timely detection of change points in the input data. The authors in [13] present a comprehensive view of mobile sensing systems (MSSs). Modern smart phones are equipped with rich-sensors to sense objects which can be people-centered or environment-centered. The MSS uses a user-level application running on smart phones for reading internal sensor data and dispatches the sensed data for further processing. The application programming interface (API) is required for a phone operating system to read and dispatch the data. The MSS can be used in various domains such as personal health care sensing, vehicular sensing, smart home sensing, and smart city sensing. However, MSSs have some social and technical limitations. The social barriers include privacy concerns and the absence of economic incentives that might encourage people to participate in a sensing campaign, while a technical barrier could be phone energy savings, limited battery life, and a variety of sensors and software for their management. In particular, the work presented in [13] is closely related to our own because MSS is used by the participants to record their activities. The API running on the phone uses the internal sensor reading for recording and reporting the user activities, and also asks the user to identify the start and end of each activity performed. However, in [13], users are required to manually review and label some or all of their performed activities offline. Our proposed approach focuses on the automatic identification of changes in user activities to facilitate the activity labeling by prompting/requesting input from users online, at the point of change.

The self-adaptive behavior-aware recruitment (SBR) scheme [14] has been used in participatory sensing to identify activities according to the participants’ behavior using sensor-enabled smart devices. The tempo-spatial behavior and data quality is evaluated by the SBR scheme for efficient data collection in participatory sensing. The SBR scheme has the advantage of stability, self-adaptiveness, and providing efficient sensing performance. The work in [14] focuses on the evaluation of recruitment strategy on participants’ selection for participatory sensing, which is an important but different aspect of sensing from our own. The five-tier participatory sensing systems (PSSs) framework [15] has been proposed and achieved better sensing coverage with a minimum number data collection points (DC-points). The PSSs framework is comprised of five layers (namely, data collection points deployment layer, participant recruitment layer, data-sensing layer, data transmission layer, and data-processing layer) and each layer has its own functionality. The first layer determines data collection points for an optimized deployment scheme in a given monitoring area. The second layer evaluates the static and dynamic deployment scheme using the Wise-Dynamic DC-points Deployment (WD3) algorithm in order to deploy the data collection points for high-quality sensing. The third layer is used to identify and sense the surrounding environment using various sensors embedded in smart devices such as smart phones, smart watches, and others. The fourth layer is used for reliable data transmission to the data center for further processing. The fifth layer is used to analyze and evaluate the transmitted data. The work in [15] focuses on better sensing coverage with a minimum number of data collection points, and again we focus on finding out the time to generate intervention to request timely labels for the most recent activities in order to generate high quality real world labeled datasets in a free living environment. Similarly, the authors in [16] have evaluated three approaches: participatory (PART), context-triggered in situ (SITU), and context-triggered post (POST). These approaches are used to record and annotate user data in real world settings. In the first approach, the participants are asked to use an interface to manually label their activities; they can start, stop, and pause the recordings. Labeling is performed offline and after the recording of the activities. In the second approach, the participant’s activities are monitored and the user is prompted to annotate their activities. Moreover, in the third approach, when the participants performed their activities, the detected activities were stored in a repository. However, a reminder is sent later to annotate the performed activities. Again, labeling is performed offline and after the recording of the activities. The study has shown that SITU and POST generate more activity recordings and PART produces a huge amount of activity recordings in terms of length. Moreover, the evaluation results have shown that the recordings of PART have less noise, and are more precise and complete than SITU and POST. However, users often are required to take control of what and when to record and annotate an activity. SITU has a similar concept as ours in terms of real time labeling followed by the completion of an activity, however, users are responsible for remembering the provision of activity labels. In contrast, in our approach, the change point detected from one activity to another can be utilized to automatically issue a prompt for users to provide the label for the activity last performed. The authors of the paper [16] discussed such limitations in their work and encouraged automated recording and reminders to ease their burden. Different approaches have been used in the literature for change-point detection in health sensor data. For example, an activity-recognition algorithm was previously used to detect changes in daily life activities with the help of a Gaussian mixture classifier [6] based on mobile data. Some activities, such as stationary and nonstationary, were classified as standing-still and running, respectively. The authors used three consecutive windows of nine seconds each in the entire activity-detection process in their proposed solution. Moreover, some activities such as stand-still and walking could be detected and labeled simultaneously at changeover points. Some of the limitations of the approach were the short delay that caused incorrect detection of user activity and unsuitability of the aforementioned technique in real-time scenarios in such situations when the user transitions from nonstationary “walking” to stationary “standing-still”. Similarly, cumulative sum control chart (CUSUM) is a technique that is effective in detecting small shifts, using the mean of the process in cardiovascular events [17]. These authors have used some core methods in order to evaluate physiological monitoring modules. The core methods are the hierarchal online activity-recognition method and the biometric extraction method. In the hierarchal online activity-recognition method, first the preprocessing is performed using a finite impulse response filter. In the second step, the fast Fourier transform (FFT) has been used to convert the signal from the time domain to the frequency domain and extract the mean and energy feature from the preprocessed data. Finally, those features having direct impact on the performance of the activity-recognition algorithm were selected. In the biometric extraction method, first the heart rate values are extracted using the echocardiogram (ECG) signal. The FFT was applied to attenuate low-frequency noise and eliminate waveform irregularities from the signal. Finally, the 2-pass filter was used to find the local maxima of the ECG signal and detect the significant R-peaks. However, CUSUM cannot detect sudden shifts in accelerometer data and is therefore ineffective for such changes. The kernel density estimator approach has been used in [18]. In this approach, the density estimation ratios have been calculated for populations of data. Furthermore, these estimation ratios were used to identify the change points in the data. This approach has the advantage of automatic model selection and the convergence property. However, the disadvantages include difficulty in calculating density estimation for high-dimensional data, which can be slow and less robust. The authors in [19] have proposed a fuzzy Bayesian change-point detection technique using the posterior probability of the current run length in time-series data. The proposed technique works in two folds. First, the fuzzy set technique is applied to cluster and transform the initial time-series data into a new time-series with a beta distribution. Secondly, the new time-series data is further used by a Bayesian change-point model to detect the change points. Then, the change points’ positions were estimated using the Metropolis-Hastings algorithm. The advantage of using this approach is that it does not require a priori knowledge of the distribution, but it is computationally expensive. Similarly, a one-class support vector machine has been used for change detection in human activities [20]. The authors used a high-dimensional hypersphere in order to model data and to evaluate the change-point detection based on the distribution of radii of hyperspheres. The high and low values correspond to changes in different activities. Event detection in human-activity monitoring can significantly reduce transmissions [21]. The transition between postures is difficult to classify and therefore remains unlabeled. The data is captured through accelerometer sensors placed on different parts of the body. Moreover, a posture-activity monitoring system has been developed that can classify posture form the observed data. The time-based filtering, a naïve voting scheme, and an exponentially weighted voting scheme have been used to improve the posture classification accuracy. The exponentially weighted voting scheme outperforms other schemes in event detection. Also, the transmission is reduced from original 10 Hz to about 600 event transmissions in 30 min. The Kullback-Leibler importance estimation procedure (KLIEP) approach has been proposed in [22] for change-point detection in time-series data. The Gaussian mean variance has been used in this approach [23] to extract features from the data and evaluate it. The approach has the advantages of convergence properties and automatic model selection. However, the limitations are that the density estimation for high-dimensional data is difficult to calculate and it is also computationally expensive. 

In summary, the analysis of the background literature reflects that the current change-point detection methods tend to be quite sophisticated in nature. In addition, multivariate data involves observation and analysis of more than one variable at the same time. Therefore, accurate change-point detection in user activity requires tuning of various parameters. Optimization is the process of fine-tuning input parameters to find the maximum or minimum output. The genetic algorithm has been used in the literature for a diverse range of optimization problems [12]. In our current work, we consider multivariate change-point detection as an area which has been neglected in the literature, and develop approaches which take account of changes in covariances of time-series data as well as other features, which can improve change-point detection.

## 3. The Proposed Model

The MEWMA approach is a statistical method that averages the input data within a data stream and assigns lower weights to earlier data points. The primary aim of using the MEWMA is to detect small shifts quickly in time-series data. In the proposed solution, the MEWMA is used to analyze all the covarying time-series data at the same time thus taking into account the interrelationship among the variables. MEWMA is used with standard and tuned parameters such as λ, which weights the current data versus historical data, window size, and statistical significance values, with the aim of accurate change-point detection. In addition, we use the GA to automatically identify an optimal parameter set for the MEWMA including λ, window size, and significance value for each activity by evaluating the fitness function of *F_measure*.

### The Multivariate Exponentially Weighted Moving Average (MEWMA) Change-Point Detection Algorithm

MEWMA averages the input data within a data stream and gives less weight to earlier data points. The primary aim of using MEWMA is to detect small shifts quickly in the data [24]. The results of the MEWMA technique rely on EWMA statistics, which is an exponentially weighted moving average of all prior data, including historical and current data. The multivariate EWMA is an extension of univariate EWMA to multivariate data [25] in order to monitor and analyze the multivariate process. The MEWMA is defined as:

(1) $$Z_{i}=\Lambda X_{i}+\left(1-\Lambda \right)Z_{i-1},i=1,2,3....n$$

where $Z_{i}$ is the *i*-th MEWMA vector, Λ is the diagonal matrix with elements $\lambda_{i}$ for *i* = 1, …, *p* and where *p* is the number of dimensions, and $0<\lambda_{i}\;\leq 1$, and $X_{i}$ is the i-th input vector, i=1,2,3…n. The out-of-control signal is defined in Equation (2)

(2) $$T_{i}^{2}=Z_{i}^{\prime }\sum_{i}^{-1}Z_{i}<h$$

where $Z_{i}$ is the MEWMA vector and $Z_{i}^{\prime }$ is its transpose. $\Sigma_{i}$ is the variance covariance matrix of $Z_{i}$ and *h* (>0), is chosen to achieve a specified in-control signal. Multivariate analysis is used to measure more than one characteristic of a system and also to evaluate the relationship among these characteristics. In multivariate analysis, we consider the data stream of length q consisting of specific data points $X_{1},X_{2},X_{3}\ldots X_{q}$ (e.g., for accelerometer value $X_{i}=\left(-1.858,-9.649,\;1.132\right)$ where the elements represent the x, y, and z values of 3-dimensional accelerometer signal). In general, a sequence of data point $X_{1}$ to $X_{q}$ may contain different distributions. In particular, the two subsequences $X_{1},X_{2},X_{3}\ldots X_{i-1}$ and $X_{i},X_{i+1}\ldots X_{q}$ may follow different distributions (say, for example, D_1_ and D_2_, where D_1_ and D_2_ can be equal or different). The aim of the algorithm is to determine and classify the position of change points $x_{i}$ in the data stream. In each data stream, MEWMA is used to evaluate the position of change points and calculate the exponentially weighted moving average of multivariate input vectors $X_{i}$ to provide accurate change-point detection. We consider a number of possible values for the window sizes (1 s, 1.5 s, 2 s, 2.5 s, 3 s), which are used to analyze the data using a sliding window with an increment of 1 data point to perform sequential analysis. The window sizes are used to evaluate the sequence from inside the window. These window sizes are chosen to combine some historical data with new data to balance the data and identify if the change happens. Also, these are reasonable sizes that are taken from experimentation. Likewise, the $Z_{i}$ represents the MEWMA vector and is calculated by using the multivariate input vectors as shown in Equation (1). In addition, the variance-covariance matrix of $Z_{i}$ is calculated recursively and represented by $\Sigma_{i}$ to find T-squared, as shown in Equation (2).

Once the T-squared statistic is calculated as shown in Equation (2), we consider a number of possible values for the significance values *h* (0.05, 0.025, 0.01, 0.005), which are used to identify the confidence of the entire window. These values are used in literature and define regions where the test statistics are unlikely to lie [26]. If the T-squared value is greater than *h*, then $x_{i}$ will be labeled as a change point within the data stream. The analysis of the accelerometer data identifies the actual values of the specific change points, which may represent an increase or decrease in the data. Thus when executing a sliding window version of the algorithm, change points are detected which are adjacent as the data points become increasingly indicative of a “significant” change. However, if the adjacent detected change points represent the same event of the real change point in the data stream, then the new parameter *k* is used to eliminate such adjacent change points.

Arguably the most significant branch of computational intelligence is evolutionary algorithms (EAs), which have much potential to be used in many application areas. The basic concepts of EAs are inspired by observing the biological structure of nature; for instance, the selection and genetic changes could be used to find the optimal solution for a given optimization problem [27]. Moreover, the robust and adaptive characteristics of EAs are performing a global search instead of a local search to find the optimal solution in the search space. The GA is a machine learning method which is inspired by the genetic and selection structure of nature [28]. Also, the predefined fitness function is optimized by performing a randomized and parallel search to find the optimal solution [29]. The GA starts with a random sample of variable sets and repeatedly modifies a population of individual solutions. Various criteria can be used for the selection process to obtain the desired solution through the evaluation of individual solutions. The best individual solution is selected as an input for the next generation. The GA is used for solving optimization problems based on natural selection, which is the process used in driving biological evolution [12]. The optimization modifies input characteristics of a system using a mathematical process to find the minimum or maximum output. The objective of the fitness function in the GA is used to find the optimal solution to a system. In our case, each distinct combination of the three variables provides a single solution in the population, namely $\lambda_{i}$, the window size, and the significance. Over a number of generations, these solutions “evolve” towards the optimal solution [30].

The fitness function is the core component of the GA. It evaluates each individual parameter set in the population to find the solution with an optimal fitness value. In our fitness function, we initialize the population of vectors whose elements contain the $\lambda_{i}$ values, the window sizes, and the significance values. Our fitness function then tries to find the solution with the maximum *F_measure* value given a range of input values. The *F_measure* is used as the measure to find the overall effectiveness of the activity recognition by combining the precision and recall. The fitness function can be defined as follows:

(3) $$F\_measure_{max}\;=\;max_{\left(\lambda ,win\_size,sig\_value\right)}\left(F\_measure_{MEWMA}\right)$$

For simplicity, we assume $\lambda_{i}$ is equal to λ for i=1,…,p, where **λ***_i_* ranges from 0.1 to 1 for each activity with the corresponding significance values of 0.05, 0.01, 0.025, 0.005 and window sizes of 1 s, 1.5 s, 2 s, 2.5 s and 3 s. Our proposed model uses Equation (3) as the fitness function by initializing upper and lower bounds of the three parameters to find the maximum *F_measure* with the optimal parameter set. After the exploration with different parameter settings, the optimal GA parameters, which maximize the fitness function of the *F_measure*, are shown in Table 1.

The selection function in the GA chooses the parents for the next generation based on their scale values by evaluating the fitness function. As we need to find the maximum value of the fitness function using Equation (3), the individual with the maximum value of the fitness function has greater chance for reproduction and also for generation of offspring. Here we used stochastic uniform to build in randomness. The reproduction function helps to determine how the GA creates children at each new generation. Elite count or the crossover fraction can be used to create new children at each generation. The first method specifies the number of individuals that are guaranteed to survive in next generation. However, the later method specifies the fraction of the next generation which crossover produces; we here use reproduction probability 0.8 and mutation with probability 0.2 so as to allow some new values to take part in the optimization process.

The crossover combines two individuals or parents to form a new individual or child for the next generation. Different methods such as constraint dependent, scattered, heuristic, and arithmetic approaches can be used depending on the problem requirement. We choose the scatter method to make random selection. In the population, the mutation function makes small random changes in the individuals, which provide genetic diversity and enable the GA to search in a broader space. Different methods can be used for this, such as the Gaussian function, uniform function, and adaptive feasible function for random modification. We choose an adaptive feasible solution because it randomly generates directions that are adaptable with respect to the last successful generation.

**Figure 1:**
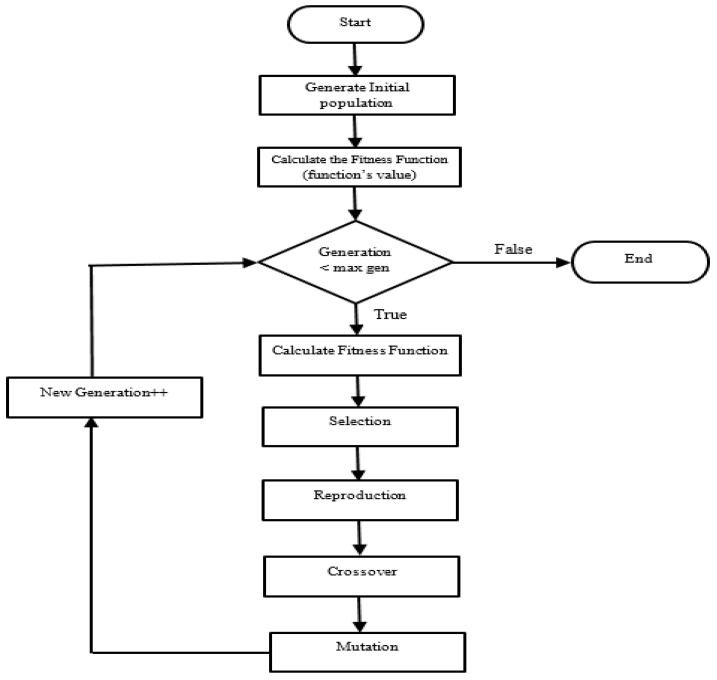
Flow chart of various stages to perform genetic algorithm (GA) optimization.

The GA process, illustrated in Figure 1 with respect to the GA parameters proposed in Table 1, is described as follows [30]:

- The population size is initialized with the number 50, which specifies how many individuals there are in each of the iterations. Usually, the number 50 is used for a problem with five or fewer variables, and the number of 200 is used otherwise.
- Check the termination condition of the algorithm on if the number of generations has exceeded the maximum value. If so, the GA algorithm is terminated, otherwise, continue with the following steps.
- Calculate the maximum value of the fitness function using Equation (3).
- The individuals are selected from the current population applying a stochastic uniform function. Each parent corresponds to a section proportional to its expectation. The algorithm moves along in steps of equal size. At each step, a parent is allocated from the section uniformly.
- The individuals are then reproduced randomly with a fraction using the crossover operation. The scatter function is used to select the genes where the vector is 1 from the first parent and 0 from the second parent before combining them to form a child.
- is then applied with the adaptive feasible method to randomly generate individuals in the population.
- Finally, a new generation is updated and the GA algorithm loops back to check the termination condition. The default value for the generations is 100 multiplied by the number of variables used, but we choose the best value for generation by experimentation with different values.

## 4. Evaluation

**Figure 2:**
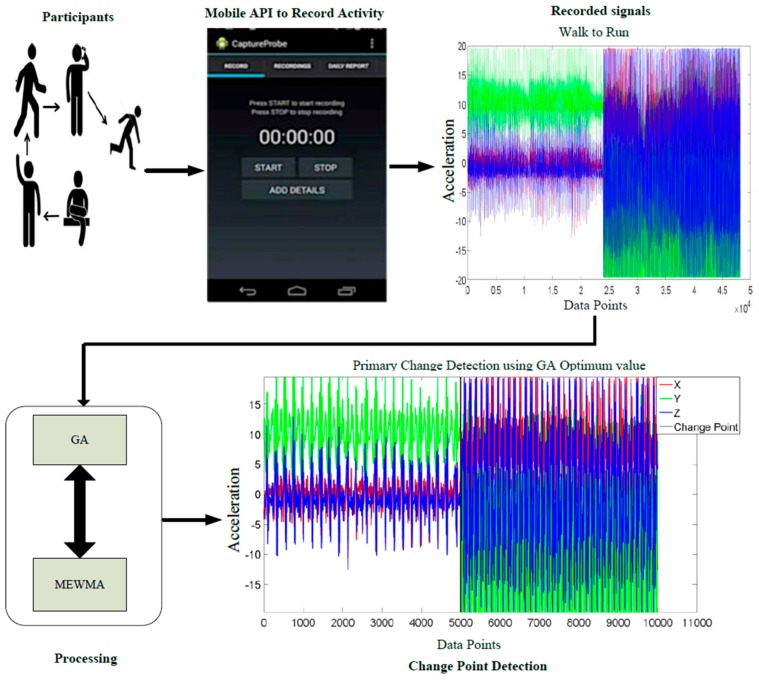
The system model.

In our experiments we used a real dataset for evaluation. AlgoSnap uses the CrowdSignals platform to collect sample datasets to help and support researchers in the academia. CrowdSignals.io is a nonprofit research community. The CrowdSignals platform was created by AlgoSnap to build a large labeled mobile and sensor dataset for the research community. Our sample dataset is taken from the above platform and fed to the algorithm as a stream, to represent a real deployment. This sample dataset was collected from two participants who kept a smartphone inside the right-front pant pocket and wore a smartwatch on the dominant wrist [31]. The data from each participant was captured continuously for 2.5 h using 20 sensors with sample frequency of 74.4 Hz. Each participant performed eight different activities and also labeled these activities. The eight different activities performed by each participant were eating, washing hands, smartphone kept on the table, sitting, standing, walking, running, and driving. The duration of an activity varied from 1 min to 5 min depending on the activity. A transition could be regarded as an activity itself, especially if takes a long time, however, here we focus on the core activities and primary change points. The time delay ranges from 5 ms to 12 ms. The participant used the smart phone Android app online to explicitly label the start and end times of each activity performed. Moreover, the labeled data is sent periodically to the server which runs the GA offline for optimization as shown in Figure 2. The start and the end time for each activity are denoted in the dataset as a truth table. In the sample dataset, various sensors were used to collect data, but only accelerometer data is used in our experiments. For illustrative purpose, only one accelerometer sensor was used, with three dimensions, but other authors have demonstrated how multimodal sensors can be used to increase activities recognition and enable the recognition of activities in various situations [32]. After the data collection, the activity execution of accelerometer data was wirelessly streamed to a receiving computer via the IEEE 802.15.1 Bluetooth communications protocol.

The study in [33] elaborates the high acceptance for telemedicine and usability of a telemedicine approach. The deployment of such an application is useful in emergency situations and achieves higher accuracy and quality of data for monitoring of patient vital parameters over time. A limitation could be the privacy issues, date security, and high probability of false alarms. In our work, we partly address the additional problem of low user acceptance due to excessive requirements to interact with the mobile phone.

### 4.1. Experimental Results

A real dataset, as described, has been used by the GA to identify the optimal set of parameters for the MEWMA approach in change-point detection. For the multivariate approach the *x*, *y* and *z* acceleration magnitude is calculated from the captured data and used as the input to the MEWMA algorithm. The MEWMA algorithm is initially used to analyze different parameters including λ (0.1 to 1), the window size (1 s, 1.5 s, 2 s, 2.5 s, 3 s) and the significance values (0.05, 0.025, 0.01, and 0.005) to find the accurate change point. We considered all the values of λ in the range varying from 0.1 to 1 to allow for some contribution from both historical data and current data. Moreover, MEWMA also combines historical data and current data. Following this, the GA is used to identify the optimal set of parameters for the MEWMA algorithm. However, the GA implemented in Matlab 2014 typically takes a long time, where in our experiments it takes approximately between 10 min and 25 min to run on a system with processor 3.40 GHz and 8 GB RAM. The parameter values are not likely to change too frequently, so the GA could be run offline periodically. The *F_measure* metric was used to evaluate the optimal change point in the activity monitoring using the GA. A detected change point is considered to be true if in the data stream the index *i*, *i* ∈ {*z* − (*f*/4),…, *z* + (*f*/4)} where *z* indicates the index of a manually labeled change in the data stream and *f* denotes the sampling frequency in Hz. In our experiment we formed a dataset containing activities such as walking to running, walking to driving, walking to washing hands, walking to standing, and walking to sitting.

**Figure 3:**
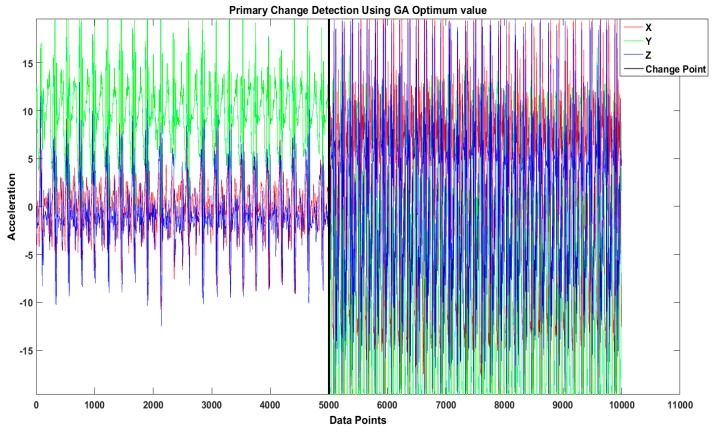
Real dataset example of sliding window change-detection result for the activity “walking to running”.

**Figure 4:**
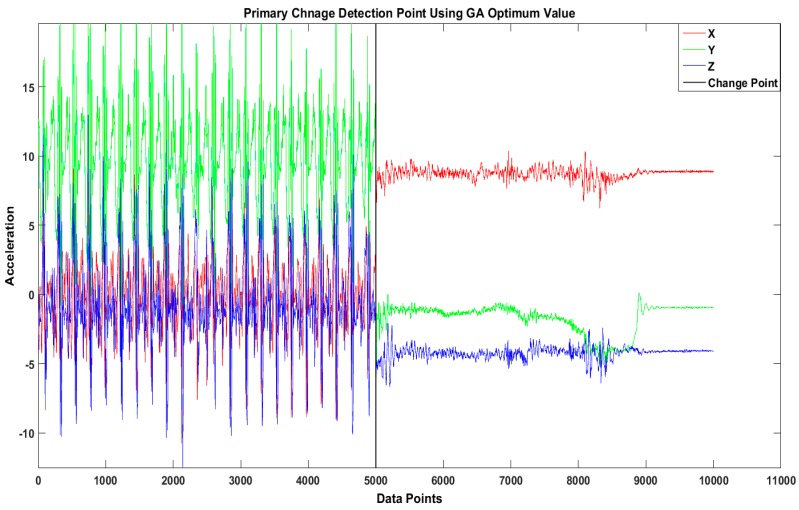
Real dataset example of sliding window change-detection results for the activity “walking to driving”.

The objective of our proposed technique is to identify the optimal set of MEWMA parameters using the GA for detecting change points in high-level activities such as walking to running and walking to driving, examples of which are shown in Figure 3 and Figure 4 respectively. The sliding window with optimal change-point detection parameters for the activity “walking to running” has window size of 3 s with significance value *p* = 0.05 and λ = 0.7. The optimal change-point detection parameters for the activity “walking to driving” are that window size is 2.5 s, significance value *p* = 0.05, and λ = 0.6.

The experimental results on real datasets of five different activities are presented in Table 2. Moreover, the experimental results identify the changes between core activities as shown in Table 2. Here, the data points relating to the core activities are used to determine when the change points occur.

In our experiments, we analyzed dynamic activities such as walking followed by another dynamic activity such as running or driving due to its complexity and varying characteristics.

The proposed approach optimized the MEWMA parameters in order to find the best set of parameters for accurate change point detection for the different activities presented in the Table 2.

Furthermore, accuracy and *F_measure* metrics have been used to find the optimal parameters selection of the MEWMA algorithm. The accuracy is the ratio of the number of correctly classified data points to the total number of data points. Accuracy can be calculated using Equation (4):

(4) $$\mathrm{Accuracy}=\frac{\mathrm{TP}+\mathrm{TN}}{\mathrm{TP}+\mathrm{TN}+\mathrm{FP}+\mathrm{FN}}$$

Precision is defined as the number of true positives (TP) over the number of true positives plus the number of false positives (FP), whereas, recall, also known as sensitivity, is defined as the number of TP over the number of TPs plus the number of false negatives (FN). The precision and recall can be calculated using Equations (5) and (6) respectively.

(5) $$\mathrm{Precision}=\frac{\mathrm{TP}}{\mathrm{TP}+\mathrm{FP}}$$

(6) $$\mathrm{Recall}=\frac{\mathrm{TP}}{\mathrm{TP}+\mathrm{FN}}$$

The *F_measure* is used to find the overall effectiveness of the activity recognition by combining precision and recall. The *F_measure* is calculated using Equation (7).

(7) $$F\_measure=\frac{2\times \mathrm{Recall}\times \mathrm{Precision}}{\left(\mathrm{Recall}+\mathrm{Precision}\right)}$$

The non-optimized experimental results on the real dataset are presented in Table 2. The maximum *F_measure* and accuracy values are in the range of 40%–50% and 98.5%–99.4%, respectively among all the activities. The walking activity followed by a static activity achieved a maximum *F_measure* of about 50%, whereas subsequent dynamic activities have achieved 40%.

However, the optimized experimental results on a real dataset that achieved the maximum accuracy and *F_measure* were in the range of 99.4%–99.8% and 50%–66.7%, respectively. The walking activity followed by static activity achieved a maximum *F_measure* of circa 66.7%, whereas subsequent dynamic activities achieved 50%.

The highest accuracy and *F_measure* values in the experimental results on real dataset are achieved using the GA optimal parameter set of λ (0.4–0.7), significance value *p* = 0.05 and window sizes (1.5 s, 2 s, 2.5 s and 3 s) as shown in Table 2.

The highest *F_measure* values achieved are 50%–66.7% for all activities using the optimal parameter set with the real dataset. A dynamic activity such as walking followed by a static activity such as sitting, standing, and hand washing achieved the highest *F_measure* of 66.7% with an optimal parameter set of λ (0.4 and 0.5), significance value *p* = 0.05, and window size 1.5 s and 2 s. However, the subsequent dynamic activities such as driving and running achieved the highest *F_measure* of 50% with an optimal parameter set of λ (0.6 and 0.7), significance value *p* = 0.05, and window size 2.5 s and 3 s. Moreover, the accuracies achieved with optimal parameter set by the GA ranged from 99.4% to 99.8% as shown in Table 2.

The experimental results show that the *F_measure* values are relatively higher using the optimal parameter set from the GA than the results with non-optimized parameters. Additionally, in Table 2, the accuracies are also improved from 98.5% to 99.4% with non-optimized parameters to 99.4% to 99.8% with the optimized parameters. When we take out the inter-activity transition period and simulate data on this basis, the advantage of using the GA optimization is even more significant. The reason is that in the simulated data we ignored the transition data, which may be from a different distribution from the data relating to the core activities [34].

### 4.2. Walking in the Wild

**Figure 5:**
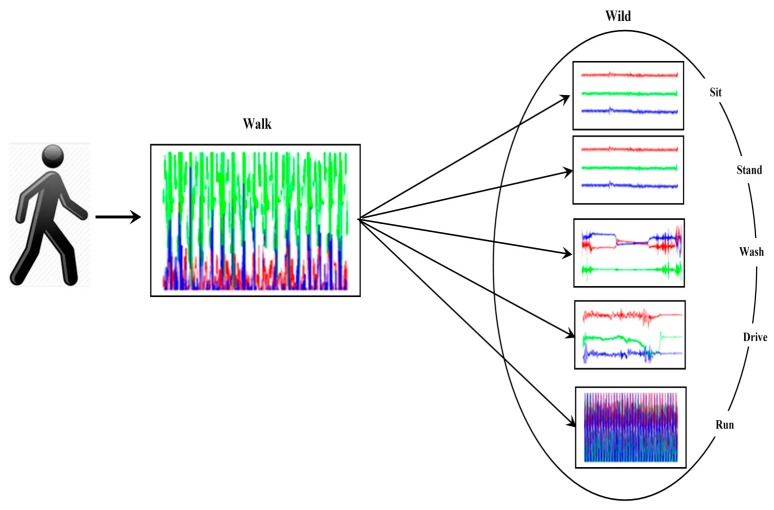
Walk to wild.

Generally, sensor data is collected in a laboratory setting and subjects perform the activities that are specified by experimenters. In the wild, however, behavior is not prescribed and the sensor data must be labeled during or after the sensor data is generated, as shown in Figure 5. This problem occurs in online change detection in real-time scenarios. In this scenario, we can alert the reminding software that we would like to sample data more frequently to increase the accuracy of activity detection. Also, we would like to be able to identify and detect early on that a change seems to be happening and ask the user for some information on what activity is actually being performed in order to improve our algorithm. An alert about the change could be issued to get a response from the user on what activity it is being performed. The alert and response thus provides more new labeled data for learning. Periodically we rerun the GA algorithm offline using new data. The data is typically processed locally on a mobile phone or smart watch but a summary of the data is transferred to the server periodically.

When the person is walking or sitting for long time, the storing or handling of the data could drain the battery as a mobile device typically has limited battery capability. The assumption of this work is that we need a lightweight and early warning indicator when a change is about to happen.

We also performed experiments on walk-to-the-wild irrespective of the activity which is happening next, as presented in Table 3. The optimal parameter set is discovered for accurate change detection using the GA. The best *F_measure* and accuracy achieved was 66.7% and 99.8% respectively with the optimal parameter set of λ = 0.7, significance value *p* = 0.05, and window size 3 s. The experimental results of walk to wild are presented in Table 3.

A class imbalance problem usually exists in datasets when the total number of instances of one class (the minority) is excessively low as compared with the number of instances of the other (majority) class [35]. This highlights the skewed distribution of classes within the dataset, and often the minority class is the class of interest [36]. In our dataset, we have only one TP point (represents a correctly identified change point) and a high number of TN (the non-transitional points which are not labeled as change). We used the *F_measure* for evaluation because it is a combination of precision and recall, as presented in Equation (7). As the precision is the ratio of TP over the total number of TP and FP (the non-transition point which the algorithm highlighted as a change) therefore one or two FP detections reduced the *F_measure* to 66.7% and 50%, respectively, due to the imbalance class problem in our real dataset.

## 5. Conclusions

This paper describes the use of a genetic algorithm to identify the optimal set of parameters for the MEWMA approach and automatically detect change points corresponding to different transitions in the user activities. The different parameters of the MEWMA are analyzed and evaluated to identify the optimal set of parameters for each activity using the GA. The optimal set of parameters selected using the GA outperformed on real world accelerometer data in terms of the accuracy and the *F_measure*. The results of the real dataset were evaluated with the optimal parameter set and improved the accuracy from 99.4% to 99.8% and *F_measure* up to 66.7%. Moreover, the MEWMA is a lightweight algorithm and can be incorporated into real world systems such as mobile-based applications for the collection and active sampling of labeled data. In the context of activity monitoring, the automatic optimization of the optimal parameter set was considered within this study. The change points in the data can be used to identify changes in activities and recognize and monitor good behavior such as healthy exercise patterns based on these activities. One limitation of this study is that a transition could be regarded as an activity in itself, especially if it takes a long time. The class imbalance problem has great impact on the classification and can be addressed using sampling-based algorithms to stabilize the majority and minority classes. Online bagging and boosting algorithms will be used in future work to tackle this imbalance class problem in the data streams. Moreover, other multivariate algorithms and optimization techniques will be explored from the state of the art literature for automatic change detection using optimal parameter selection. Also, in the future different datasets will be used for evaluation with multiple change points for complex user activities.

## Figures and Tables

**Table 1:** Genetic algorithm (GA) parameters.

Parameters	GA
Population Size	50
Selection	Stochastic uniform
Reproduction	0.8
Crossover	Scattered
Mutation	Adaptive feasible
Generations	100

**Table 2:** Non optimized and optimized with GA parameter set for five different activities on a real dataset.

Change	Sig Value	Non-Optimized				Optimized with GA			
		λ	Win Size	*F_Measure*	Accuracy	λ	Win Size	*F_Measure*	Accuracy
Walk to Sit	0.05	0.3	2 s	50%	99.4%	0.4	1.5 s	66.7%	99.8%
Walk to Stand			2 s	50%	99.4%	0.4	1.5 s	66.7%	99.8%
Walk to wash hands			2.5 s	50%	99.4%	0.5	2 s	66.7%	99.8%
Walk to Driving			3 s	40%	98.5%	0.6	2.5 s	50%	99.4%
Walk to Running			3 s	40%	98.5%	0.7	3 s	50%	99.4%

**Table 3:** Optimized parameter set with GA for walk to wild on real dataset.

Activity	λ	Win Size	Sig Value	*F_Measure*	Accuracy
Walk to Wild	0.7	3 s	0.05	66.7%	99.8%
